# A case of minimal change disease after the administration of anti receptor activator of nuclear factor kappa B ligand (RANKL) monoclonal antibody: a case report

**DOI:** 10.1186/s12882-020-02066-3

**Published:** 2020-09-29

**Authors:** Keisuke Horikoshi, Norihiko Sakai, Naoki Yamamoto, Hisayuki Ogura, Koichi Sato, Taro Miyagawa, Shinji Kitajima, Tadashi Toyama, Akinori Hara, Yasunori Iwata, Miho Shimizu, Kengo Furuichi, Takashi Wada

**Affiliations:** 1 Department of Nephrology and Laboratory Medicine, Institute of Medical, Pharmaceutical and Health Sciences, Kanazawa University, 13-1 Takaramachi, Kanazawa, Ishikawa 920-8641 Japan; 2 Division of Blood Purification, Kanazawa University Hospital, 13-1 Takaramachi, Kanazawa, Ishikawa 920-8641 Japan; 3 Department of Nephrology, Kanazawa Medical University School of Medicine, 1-1 Daigaku, Uchinada, Kahoku, Ishikawa 920-0293 Japan

**Keywords:** Minimal change disease, Nephrotic syndrome, RANK, RANKL, Case report

## Abstract

**Background:**

Minimal change disease (MCD) is one of the causes of idiopathic nephrotic syndrome in adults. The pathogenesis of proteinuria in MCD has not been fully understood. Recently, it has been reported that the receptor activator of nuclear factor-kappa B (RANK)/RANK ligand (RANKL) may contribute to the podocyte biology in kidney diseases. Denosumab is a human anti-RANKL monoclonal antibody used to treat osteoporosis. Here we report a case of MCD after denosumab administration.

**Case presentation:**

A 59-year-old male without any episodes of proteinuria was given denosumab to treat osteoporosis. Two weeks after its administration, he noticed a foamy urine and bilateral pretibial edema. Laboratory tests revealed that he had severe proteinuria (15g/g Cr), hypoproteinemia (4.0g/dL), and hypoalbuminemia (1.5g/dL). Based on the results, he was diagnosed with nephrotic syndrome. The proteinuria selectivity index was 0.05, indicating selective proteinuria. Renal biopsy showed minor glomerular abnormality with less tubulointerstitial damage, and electron microscopy showed extensive foot process effacement, indicating MCD. With all these results, glucocorticoid therapy of 50mg/day prednisolone was started. After 4weeks of treatment, the urinary protein level remains high (3.1g/g Cr). Prednisolone therapy was continued, and the levels of proteinuria decreased gradually to the range of partial remission (1.2g/g Cr) with another 7weeks of prednisolone treatment, but complete remission was not achieved.

**Conclusions:**

This might be a case wherein RANKL inhibition is associated with the pathogenesis of MCD. Further studies will be needed to elucidate the causal relationship of RANK-RANKL signaling to the pathogenesis of MCD.

## Background

Minimal change disease (MCD) is one of the causes of idiopathic nephrotic syndrome in adults, accounting for approximately 10–15% of all cases [1, 2]. Most of them respond to steroid treatment, so it has been called steroid-sensitive nephrotic syndrome. However, the response rate in adults is lower, with 5–30% of MCD adult patients not responding to initial steroid therapy [3, 4]. The pathogenesis of proteinuria in MCD has not been fully understood. Recently, it has been reported that podocytes play a key role in the mechanism of proteinuria.

The receptor activator of nuclear factor-kappa B (RANK) and its ligand RANKL are important regulators of bone mineral density [5]. RANK/RANKL is expressed not only in bone marrow-derived cells but also in non-bone marrow-derived cells such as skin epithelial cells, mammary epithelial cells, and renal glomeruli [6–10]. However, the precise role of RANK/RANKL signaling has not been known in proteinuric kidney diseases.

Denosumab is a human anti-RANKL monoclonal antibody used to treat osteoporosis [11]. Here we report a case of MCD after denosumab administration. MCD has not been reported as a side effect of denosumab.

## Case presentation

A 59-year-old male with a history of dyslipidemia and without any episodes of proteinuria was diagnosed with osteoporosis when he had knee bone fracture, the cause of which was suspected to be hypogonadotropic hypogonadism due to lower levels of luteinizing hormone, follicle-stimulating hormone, and testosterone. Other pituitary hormones were normal, with the magnetic resonance imaging showing a normal pituitary gland. He did not want to receive androgen replacement therapy, so denosumab with eldecalcitol was given to treat osteoporosis. Two weeks after its administration, he noticed a foamy urine and bilateral pretibial edema, which did not improve spontaneously. Two weeks later, he was admitted to the previous hospital, and laboratory tests showed that he had severe proteinuria (15g/g Cr) and hypoalbuminemia (1.5g/dL). Based on the results, he was diagnosed with nephrotic syndrome. He was then referred to our hospital for further examination and treatment.

Upon admission to our hospital, his height and body weight were 170cm and 65.0kg, respectively, and his blood pressure, heart rate, and body temperature were 150/86mmHg, 90bpm, and 36.3°C, respectively. Physical examination revealed bilateral pretibial pitting edema. Laboratory data for urine tests were as follows (normal ranges in parentheses): protein levels 11.7g/g Cr (<0.15), occult blood 3+ (−), red blood cells 10–19 /high power field (<5). The proteinuria selectivity index was 0.05, indicating selective proteinuria. Serum examination findings were as follows (normal ranges in parentheses): urea nitrogen 15mg/dL (7–23), serum creatinine 0.61mg/dL (0.6–1.0), total protein 4.0g/dL (6.7–8.3), serum albumin 1.5g/dL (4.0–5.0), total cholesterol 376mg/dL (128–219), triglyceride 277mg/dL (30–149), HDL cholesterol 50mg/dL (40–99), LDL cholesterol 271mg/dL (40–119), C-reactive protein 1.0mg/dL (<0.3), IgG 453mg/dL (870–1700), IgA 299mg/dL (110–410), IgM 67mg/dL (33–190), and IgE 147IU/mL (<250). Antinuclear antibody, PR3-ANCA, MPO-ANCA, and anti-GBM antibody were not detected (Table 1). Chest X-ray showed neither cardiac dilatation, pleural fluid nor abnormal shadow. The electrocardiogram showed a left axis deviation. Computed tomography showed a normal-sized kidney with a smooth surface and no mass. Gastrointestinal endoscopy showed no tumor lesion.

**Table 1:** Laboratory data on admission

**Urinalysis**		
Protein	11.7	g/g Cr
Selectivity Index	0.05	
Occult blood	(3+)	
RBC	10-19	/HPF
WBC	1-4	/HPF
Squamous cell	<1	/HPF
Transitional cell	(-)	
Tubular epithelial cell	5-9	/HPF
Hyaline cast	1+	
Epithelial cast	1+	
Granular cast	(-)	
Waxy cast	(-)	
Fatty cast	(-)	
RBC cast	(-)	
WBC cast	(-)	
**Blood count**		
WBC	7.60×10^3^	/μL
RBC	4.27×10^6^	/μL
Hb	12.7	g/dL
Ht	37.8	%
Plt	19.5×10^4^	/μL
**Biochemical test**		
TP	4.0	g/dL
Alb	1.5	g/dL
AST	22	IU/L
ALT	13	IU/L
LD	290	IU/L
γ-GTP	26	IU/L
ALP	245	IU/L
T-Bil	0.3	mg/dL
CK	164	IU/L
UN	15	mg/dL
Cr	0.61	mg/dL
UA	7.1	mg/dL
Na	143	mEq/L
K	3.5	mEq/L
Cl	113	mEq/L
Ca	6.7	mg/dL
IP	2.5	mg/dL
BS	131	mg/dL
HbA1c	5.3	%
T-Cho	376	mg/dL
TG	277	mg/dL
HDL-Cho	50	mg/dL
**Immunological test**		
CRP	1.0	mg/dL
IgG	453	mg/dL
IgA	299	mg/dL
IgM	67	mg/dL
IgE	147	IU/mL
C3	137	mg/dL
C4	41	mg/dL
CH50	60	
ANA	(-)	
MPO-ANCA	<1.0	U/mL
PR3-ANCA	<1.0	U/mL
GBM	<2.0	U/mL
**Infection**		
HBs-Ag	(-)	
HBs-Ab	(-)	
HBc-Ab	(-)	
HCV-Ab	(-)	

**Fig. 1:**
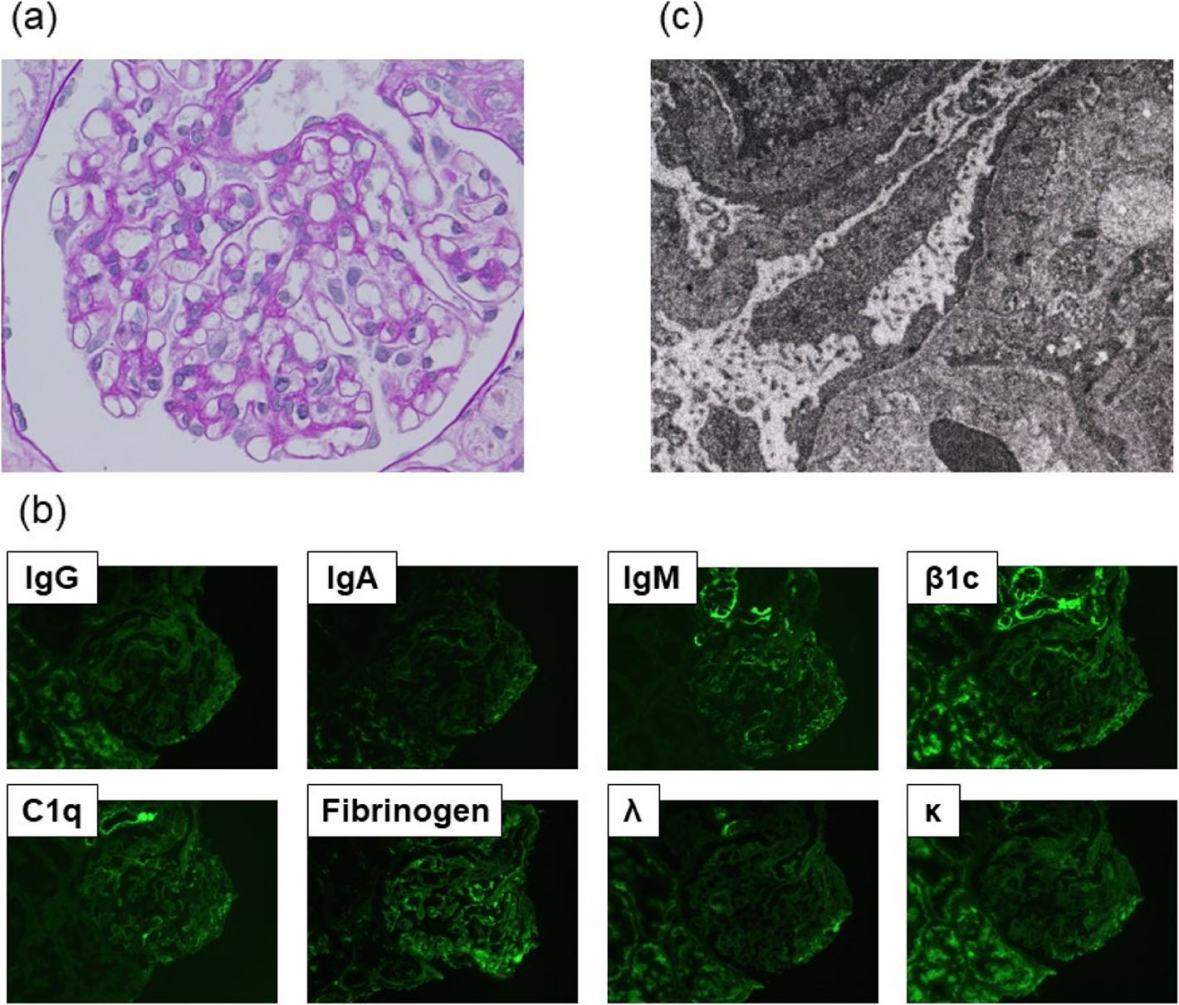
Histopathological images of renal biopsy. **a** Light microscopy showed minor glomerular abnormality with less tubulointerstitial damage (PAS staining×400). **b** Immunofluorescence studies showed no deposition. **c** Electron microscopy showed extensive foot process effacement

Upon admission, we started telmisartan treatment (40mg/day), but the level of proteinuria was not reduced. On the 7th day of admission, renal biopsy was performed to identify the cause of nephrotic syndrome. Light microscopy showed minor glomerular abnormality with less tubulointerstitial damage (Fig. 1a), immunofluorescence studies showed no deposition (Fig. 1b), and electron microscopy showed extensive foot process effacement (Fig. 1c). From these findings and clinical course, he was diagnosed with MCD. To check the glomerular expression of RANK, we tried immunostaining by anti-RANK antibody. However, it was technically difficult because there was not enough amount of section left for immunostaining.

**Fig. 2:**
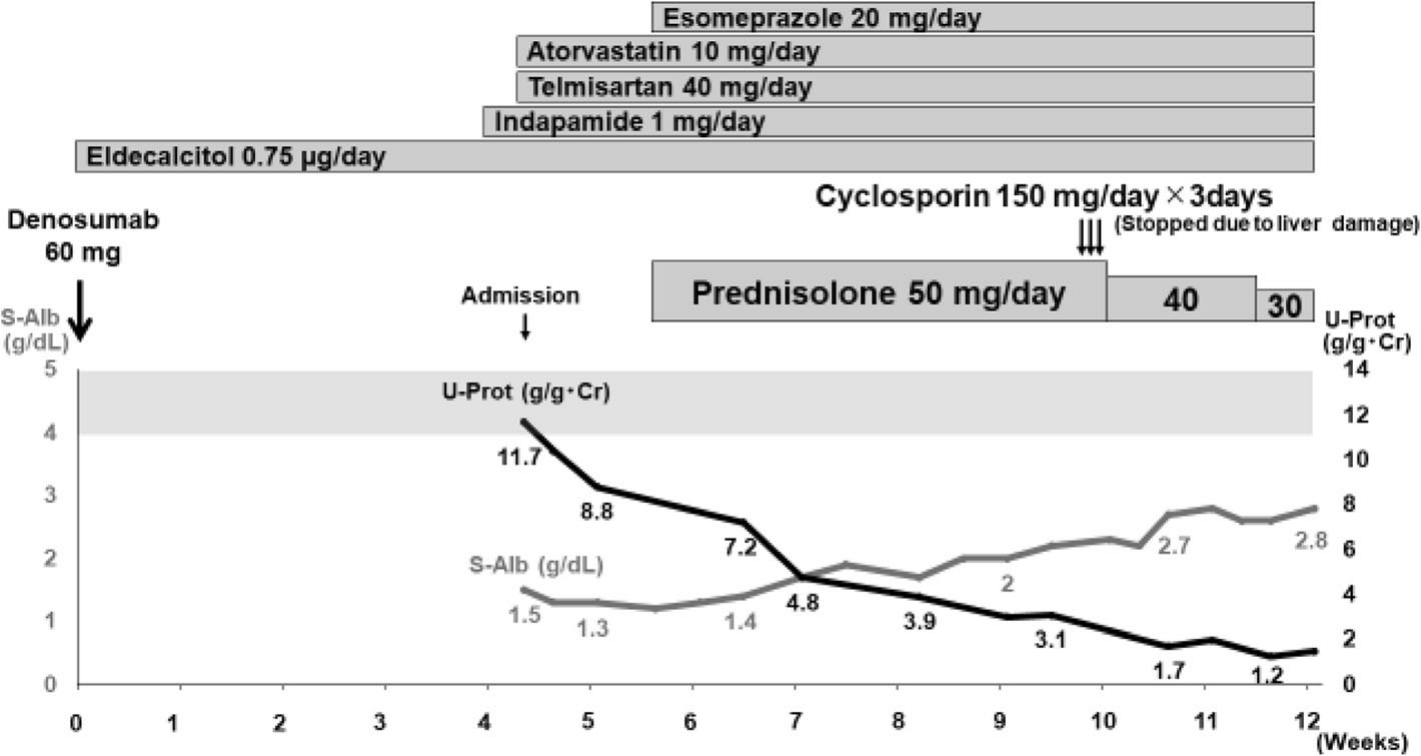
Clinical course of the case. The shaded area on the graph represents normal serum albumin level range

With all these results, glucocorticoid therapy of 50mg/day prednisolone was started. After 4weeks of treatment, the urinary protein level remains high (3.1g/g Cr). Therefore, 150mg/day cyclosporine was added but terminated due to liver damage. Prednisolone therapy was continued, and the levels of proteinuria decreased gradually to the range of partial remission (1.2g/g Cr) with another 7weeks of prednisolone treatment (Fig. 2).

## Discussion and conclusions

A 59-year-old male developed nephrotic syndrome within 2weeks after denosumab administration. From the clinical course and renal biopsy, he was diagnosed with MCD. Even with the glucocorticoid treatment, he could not achieve complete remission. We speculated that RANKL inhibition by denosumab might be involved in the onset of steroid-resistant nephrotic syndrome.

RANK and RANKL are important regulators of bone mineral density. RANK/RANKL signaling activates a variety of downstream signaling pathways required for osteoclast development, such as nuclear factor-kappa B (NF-B), mitogen-activated protein kinase, and nuclear factor of activated T cells calcineurin-dependent 1 (NFATc1) [12, 13]. RANK/RANKL is expressed not only in bone marrow-derived cells but also in non-bone marrow-derived cells including podocytes [6–10]. RANK/RANKL expression in podocytes has increased in puromycin aminonucleoside nephrosis (PAN), a rat model of podocyte injury and human kidney diseases, such as IgA nephropathy, membranous nephropathy, and focal segmental glomerulosclerosis (FSGS) [10]. In PAN rat model, the level of apoptosis of podocytes was also increased in RANK knockdown by RANK siRNA and was protected by exogenous RANKL exposure, indicating that RANK/RANKL signaling may function as the survival signal for podocytes in PAN [10]. On the other hand, lithium chloride intervention attenuated urinary protein and histopathological change by reducing the levels of RANK, RANKL, and NF-B [14]. In addition to that, Chen et al. reported that RANK and RANKL were overexpressed in the kidneys of db/db mice, a type 2 diabetic nephropathy model, and irbesartan also attenuated urinary protein by downregulating RANK/RANKL signaling and downstream NF-B pathway [15]. In this case, we tried immunostaining by anti-RANK antibody to elucidate the contribution of RANK/RANKL signaling. However, it was technically difficult because there was not enough amount of section left for immunostaining. With these results, RANK/RANKL signaling may have some contribution to the pathogenesis of proteinuric kidney diseases including MCD. To clarify the precise mechanisms of RANK/RANKL signaling in kidney diseases, further studies will be required. Furthermore, regular follow-up of urine test may be needed when denosumab is administered for osteoporosis in patients with existing renal disease and CKD.

As described above, this case showed sustained proteinuria even after steroid and cyclosporine treatment. In a previous report, the observed median T max was 10days, and T 1/2 was 25.4days following the administration of 60mg denosumab [16–18]. Therefore, the extended half-life of denosumab might cause a continuous decline of survival signal in podocytes, resulting in persistent proteinuria in this case. Additionally, because it is sometimes difficult to distinguish between MCD and FSGS, this case may have been FSGS. The accumulation of cases of nephrotic syndrome after denosumab treatment will be required to evaluate the association of RANK/RANKL signaling with treatment resistance in nephrotic syndrome.

In summary, the case of MCD after denosumab administration might be associated with RANKL inhibition. Further studies will be needed to elucidate the causal relationship of RANK/RANKL signaling and the pathogenesis of MCD.

## Data Availability

Not applicable.

## Abbreviations

MCD: Minimal change disease
RANK: Receptor activator of nuclear factor-kappa B
RANKL: Receptor activator of nuclear factor-kappa B ligand
NF- 휅B: Nuclear factor- kappa B
NFATc1: Nuclear factor of activated T cells calcineurin-dependent 1
PAN: Puromycin aminonucleoside nephrosis
FSGS: Focal segmental glomerulosclerosis
